# Regulation of enzymatic reactions by chemical composition of peptide biomolecular condensates

**DOI:** 10.1038/s42004-024-01174-7

**Published:** 2024-04-20

**Authors:** Rif Harris, Shirel Veretnik, Simran Dewan, Avigail Baruch Leshem, Ayala Lampel

**Affiliations:** 1 Shmunis School of Biomedicine and Cancer Research, George S. Wise Faculty of Life Sciences, Tel Aviv University, Tel Aviv, Israel; 2 Center for Nanoscience and Nanotechnology Tel Aviv University, Tel Aviv, 69978 Israel; 3 Sagol Center for Regenerative Biotechnology Tel Aviv University, Tel Aviv, 69978 Israel; 4 Center for the Physics and Chemistry of Living Systems Tel Aviv University, Tel Aviv, 69978 Israel

**Keywords:** Supramolecular chemistry, Nanobiotechnology, Bioinspired materials, Enzymes

## Abstract

Biomolecular condensates are condensed intracellular phases that are formed by liquid-liquid phase separation (LLPS) of proteins, either in the absence or presence of nucleic acids. These condensed phases regulate various biochemical reactions by recruitment of enzymes and substrates. Developments in the field of LLPS facilitated new insights on the regulation of compartmentalized enzymatic reactions. Yet, the influence of condensate chemical composition on enzymatic reactions is still poorly understood. Here, by using peptides as minimalistic condensate building blocks and β-galactosidase as a simple enzymatic model we show that the reaction is restricted in homotypic peptide condensates, while product formation is enhanced in peptide-RNA condensates. Our findings also show that condensate composition affects the recruitment of substrate, the spatial distribution, and the kinetics of the reaction. Thus, these findings can be further employed for the development of microreactors for biotechnological applications.

## Abstract (web summary)

Peptide condensates are known to regulate compartmentalized enzymatic reactions, however, the influence of condensate chemical composition on enzymatic reactions is still poorly understood. Here, the authors study β-galactosidase as a simple enzymatic model and reveal that product formation is enhanced in heterotypic peptide-RNA condensates, but the reaction is restricted in homotypic peptide condensates.

## Introduction

In living cells, biomolecular reactions occur in defined compartments, i.e., organelles. The majority of cellular organelles are bound by a semi-permeable lipid membrane, while others are membraneless, separated from the cytoplasm by the formation of liquid-like, condensed phases^1^. These membraneless organelles, or biomolecular condensates, are formed by various intermolecular interactions between proteins with intrinsically disordered regions, either alone, or in complexation with nucleic acids, which collectively result in liquid-liquid phase separation (LLPS). These attractive forces include electrostatic interactions, hydrogen bonding, and π interactions^1–5^. Biomolecular condensates that are formed by LLPS of a single protein as the main building block are typically termed homotypic, while condensates that are formed by complexation of various proteins, or proteins with nucleic acids are termed heterotypic. Examples of homotypic condensates include the mussel foot protein condensates, which function as a biological glue^6^, histidine-rich beak protein condensates, which are secreted from the beak of the jumbo squid^3,7,8^, and tau protein condensates, which promote polymerization of microtubules by associating with tubulin and attenuating its disassembly rate^8,9^. Yet, the majority of biomolecular condensates are heterotypic^1,10,11^, including the nucleolus, which is responsible for pre-rRNA processing and ribosomes biogenesis, polymeric leukemia nuclear bodies condensates, which are responsible for SUMOylation, and Cajal bodies, which are responsible for snRNA modification^1,10^, to name a few.

Previous studies reported on the utilization of designed condensates for regulation of various enzymatic reactions, both in vitro and in vivo^12–15^. These studies showed that biomolecular condensates can recruit specific enzymes and substrates, owing to their physical and material properties^3^. The confinement and crowding in the condensates, which increase the encounter rate of enzymes and their substrate, may result in acceleration of the reaction rate^11,12,16–23^, while limited partitioning and recruitment of specific enzymes and substrates may result in restriction of the rate of some biochemical reactions^11,12,24–26^. Yet, it is still not fully understood how the properties of condensates, and specifically their chemical composition, affect the acceleration or inhibition of reaction kinetics.

We sought to gain insights on the influence of condensate composition, and specifically the difference between homotypic and heterotypic condensates and their compositions on enzymatic reactions. For this, we utilized designed peptide condensates as a minimalistic model system of biological condensates. Peptides have recently emerged as promising building block for construction of synthetic condensates^3,5,8,14,27–31^ due to their relatively easy synthesis and purification process and lack of secondary structure. Using a designed 14-mer LLPS-promoting peptide^27^ as the primary building block, we studied three different condensate compositions: homotypic condensates that are formed by peptide simple coacervation, heterotypic condensates that are formed by complex coacervation of oppositely charged peptides, and heterotypic condensates that are formed by peptide-RNA complexation. Our findings with β-galactosidase (β-gal) as a simple enzymatic model system show dramatic differences in reaction kinetics between the different condensate systems, where the homotypic condensates restrict the reaction while the heterotypic peptide-RNA condensates accelerate reaction rate. Importantly, we show that the condensates spatially regulate the enzymatic reaction, which occurs in the condensed phases rather than the dilute phase. These insights can shed light on how biological condensates spatially regulate biochemical reactions. Furthermore, the conclusions from the work can facilitate the construction of microreactors for selective enhancement/restriction of specific enzymatic pathways.

## Results and discussion

### Reaction kinetics in homotypic and heterotypic peptide-based condensates

**Fig. 1:**
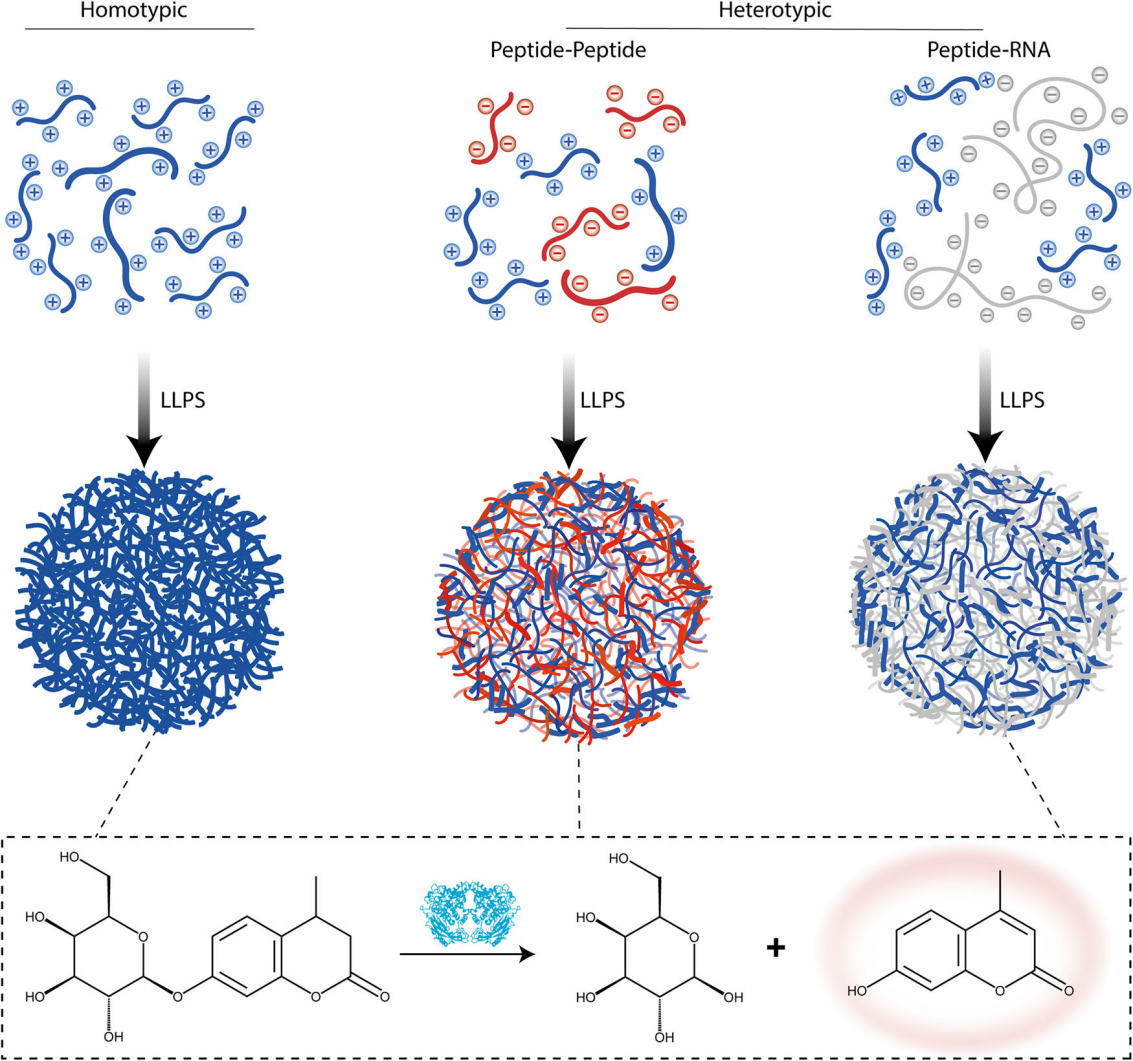
Regulation of enzymatic reactions in homotypic vs. heterotypic condensates. Schematic illustration showing the three condensate model systems (from left to right): homotypic condensate formed by peptide LLPS, heterotypic condensate formed by peptide-peptide LLPS, and heterotypic condensate formed by peptide-RNA LLPS. β-gal-catalyzed hydrolysis of 4-MUG to the fluorogenic product 4-MU is performed in each of the condensate systems.

To gain insights into how condensate composition affects biocatalysis, we analyzed three minimalistic peptide condensate systems: (i) homotypic peptide condensates, (ii) heterotypic peptide-peptide condensates, and (iii) heterotypic peptide-RNA condensates (Fig. 1). The primary building block of these condensates is a minimalistic 14-mer LLPS-promoting peptide (Fig. S1a), previously reported by us^27^. The peptide contains three repeats of arginine-glycine (RG) dyad, three aromatic amino acids, and elastin-like polypeptide (ELP) motif, which promotes phase separation^27^. As a model system for enzymatic reaction, we selected the simple and well characterized hydrolysis of terminal non-reducing β-d-galactose by the enzyme β-galactosidase (β-gal). As a substrate for the reaction, we selected the relatively hydrophobic (LogP = −0.13) substrate 4-methylumbelliferyl β-d-galactopyranoside (4-MUG). Hydrolysis of 4-MUG results in the hydrophobic (LogP = 1.89) fluorescent product 4-methylumbelliferone (4-MU) (Fig. 1), which can be easily tracked both spectroscopically and microscopically, and thus enables a spatial analysis of the reaction in condensates.

First, we characterized the condensates from each system. For all three types of condensates, the peptide(s) are dissolved in phosphate buffer at pH 7.5. The homotypic condensates are formed by peptide simple coacervation (20 mM) in the presence of 0.1 M NaCl, which is required for LLPS through charge screening of the cationic peptide^27,28^. The heterotypic peptide-peptide condensates are formed by complex coacervation of the cationic peptide and its anionic peptide counterpart (Fig. S1b), termed WGE, which contains three Gly/glutamic acid substitutions (Gly/Glu) and thus has a net charge of (−3). The peptides undergo LLPS at 1:1 stoichiometry at a final concentration of 5 mM. The heterotypic peptide-RNA condensates are formed by complex coacervation of the cationic peptide (2 mM) and poly-U (1 mg ml^−1^), which is used as a model for unstructured RNA^29,30,32^. Microscopy analysis shows that the homotypic condensates are less abundant than the heterotypic condensates, yet no significant difference in condensate size is observed between the different systems (Fig. S2).

**Fig. 2:**
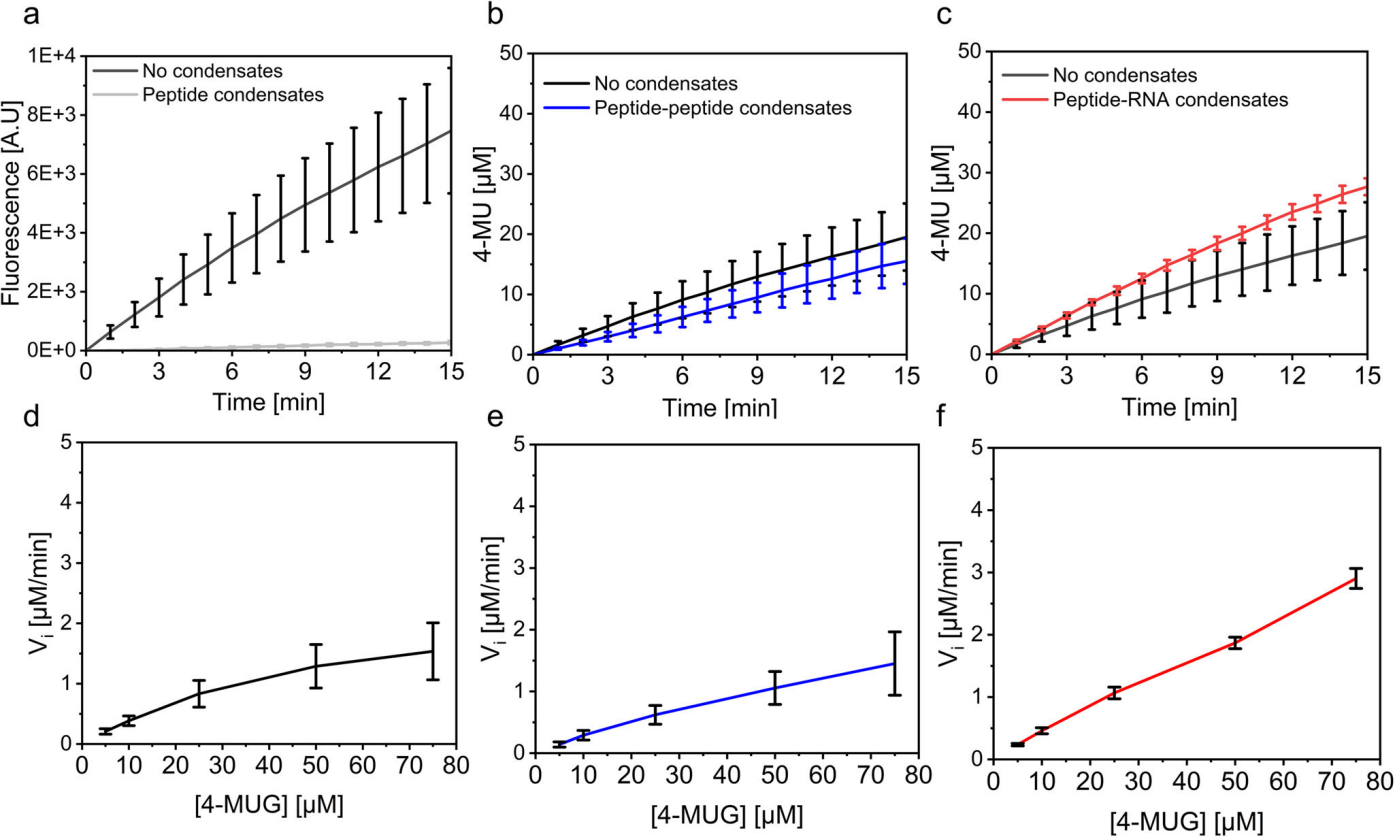
Hydrolysis of 4-MUG in designed biomolecular condensates. Fluorescence spectroscopy analysis of product formation over time (*λ*_ex_ = 320 nm and *λ*_em_ = 450 nm). **a**–**c** Kinetics of β-gal-catalyzed 4-MUG hydrolysis in the absence of condensates (black line) or in homotypic (**a**, gray line), heterotypic peptide-peptide (**b**, blue line) or peptide-RNA (**c**, red line) condensates. **d**–**f** Initial reaction rate of **d** the free enzyme, **e** enzymatic reaction in peptide-peptide condensates, and **f** enzymatic reaction in peptide-RNA condensates as a function of substrate concentration. Values represent an average of three independent measurements; error bars represent SD. All measurements represent the total contribution of the dilute and condensed phases.

Next, we monitored the enzymatic hydrolysis of 4-MUG in the different condensate systems. We expected that the hydrophobic 4-MUG substrate will partition in the condensed phase due to the difference in polarity between the dense and dilute phases, and that the negatively charged enzyme (pI = 4.61) will strongly partition in the condensates due to interactions with the cationic peptide. Taking this into account, we hypothesized that the reaction will mostly occur inside the condensates. Based on previous studies which show acceleration of enzymatic reactions in condensates^16–23^ we expected that the compartmentalization of the reactions in the condensates will accelerate the reaction rate. Before monitoring the kinetics of β-gal, we first analyzed the fluorescence of the free 4-MU product in the absence of condensates and compared it to the fluorescence of 4-MU in condensates. Notably, we found that the fluorescence of 4-MU is quenched in all condensates (Fig. S3). Thus, compared with the free 4-MU, the fluorescence intensity of the product is 5-fold, 2.5-fold, and 1.7-fold lower in the homotypic, peptide-peptide and peptide-RNA condensates, respectively (Fig. S3). We also confirmed that the condensates do not promote a spontaneous hydrolysis of 4-MUG and that the substrate hydrolysis is completely mediated by β-gal activity (Fig. S4). To analyze the enzymatic activity of β-gal in condensates, the enzyme was added to pre-formed condensates at a final concentration of 1 µg ml^−1^ (1.92 nM). After 10 min incubation, 4-MUG was subsequently added at a final concentration of 50 µM. The kinetics of the reactions was analyzed by monitoring the 4-MU product fluorescence by fluorescence spectroscopy at *λ*_ex_ = 320 nm and *λ*_em_ = 450 nm. Surprisingly, the enzymatic activity in the homotypic, but not in the heterotypic condensates, is restricted (Fig. 2a). Considering the differential quenching of the 4-MU in the different condensates, we created separate calibration curves of the product in each heterotypic condensate systems (Fig. S5) to analyze the kinetics as a function of product concentration over time. The kinetics analysis shows that the initial rate of the reaction is slower in the peptide-peptide heterotypic system than that of the free enzyme (Fig. 2b). When changing the charge ratio of the peptide-peptide condensates from 1:1 to 2:1 positive:negative charge, the reaction rate decreases by 40% (Fig. S6). In contrast, the initial rate and conversion in the peptide-RNA condensates are higher than those of the free enzyme in the absence of condensates (Fig. 2c, Supplementary Data 1). We further analyzed the enzymatic reaction by analyzing the initial rate of the reaction at varying substrate concentration, ranging between 5 μM–75 μM. Due to the poor solubility of 4-MUG we could not use substrate concentration >75 μM. Using the Michaelis–Menten model and by plotting the data using the Lineweaver-Burk analysis (Fig. S7), we calculated the *V*_max_, *K*_cat_, and *K*_m_ for each system. As presented in Table S1, the *K*_cat_ of the reaction in the peptide-peptide condensates is 1.6-fold larger than that of the free enzymatic reaction, while the *K*_cat_ of the reaction in the peptide-RNA condensates is 3.3-fold larger than that of the free enzyme. Yet, the *K*_m_ of the reactions in peptide-peptide and peptide-RNA condensates is ~2.4-fold and 3-fold higher than that of the free enzyme, respectively. Thus, the catalytic efficiency (*K*_cat_/*K*_m_) of the three systems are of the same magnitude, 3.86 ± 0.27(×10^11^)sec^−1^, 2.54 ± 1.32(×10^11^)sec^−1^, and 4.20 ± 0.92(×10^11^) sec^−1^ for the free enzyme, reaction in peptide-peptide and peptide-RNA condensates, respectively. To shed light on the restricted reaction in the homotypic condensates, we analyzed the reaction in the presence of sub-saturation concentration of the cationic peptide (1 mM). This analysis shows that even in the absence of condensates, the peptide restricts the reaction, as 23% decrease in product fluorescence is observed (Fig. S8). In contrast, no decrease in product fluorescence is observed in reaction containing 1 mM of the negatively charged peptide counterpart, WGE (Fig. S1b). This result suggests that direct interactions between the positively charged V1 peptide and the negatively charged enzyme interfere with the activity of β-gal.

### Partitioning of reaction components in condensates

**Fig. 3:**
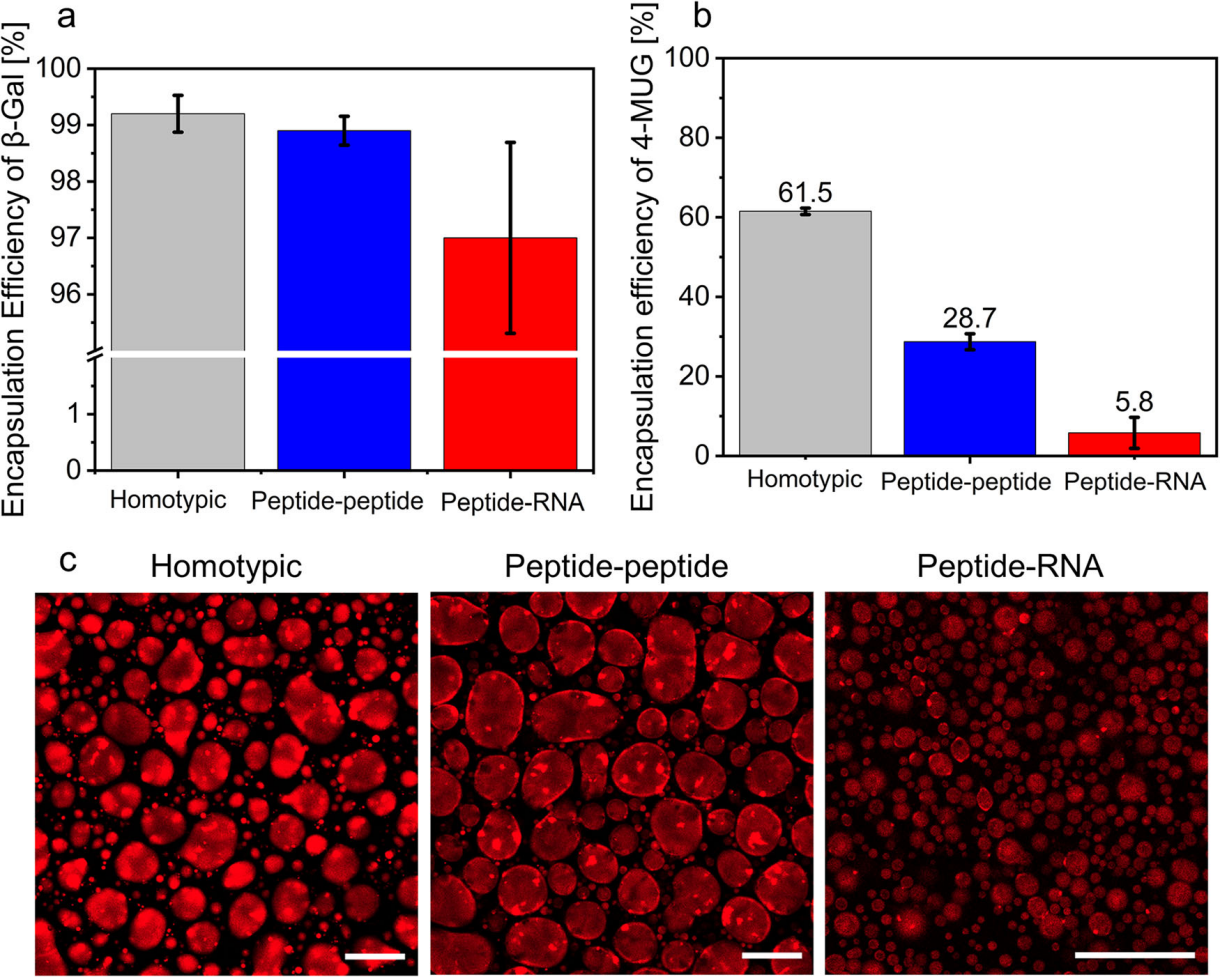
Condensate composition affects recruitment of substrate and enzyme. **a**, **b** Encapsulation efficiency (EE) of Atto633-labeled β-gal and 4-MUG in either homotypic (gray), heterotypic peptide-peptide (blue) or peptide-RNA (red) condensates. EE of Atto633-β-gal was analyzed using confocal microscopy analysis at *λ*_ex_ = 640 nm (**a**), and the EE of 4-MUG was analyzed by fluorescence spectroscopy at *λ*_ex_ = 315 nm and *λ*_em_ = 370 nm (**b**). Values represent an average of 3 independent measurements; error bars represent SD. **c** Confocal microscopy images of encapsulated Atto633-β-gal in the different condensate systems obtained using *λ*_ex_ = 640 nm. Scale bars = 50 µm.

Next, we analyzed the encapsulation efficiency (EE) of the enzyme and substrate in each type of condensates. To obtain the %EE for each condensate system, we labeled the enzyme with Atto633 and analyzed the fluorescence intensity of Atto633-β-gal in the condensed/dilute phase by using confocal microscopy. The confocal microscopy analysis shows that all three types of condensates have high %EE of 99.2 ± 0.3%, 98.9 ± 0.3%, and 97 ± 1.7% for the homotypic peptide, heterotypic peptide-peptide, and peptide-RNA condensates, respectively (Fig. 3a, c). Notably, the distribution of the labeled enzyme in all three types of condensates is inhomogeneous (Fig. S9), presumably due to aggregation of either the enzyme or the labeling dye. We analyzed the EE of 4-MUG (Fig. 3b, Supplementary Data 2) by measuring the concentration of the substrate in the dilute and condensed phase following centrifugation using fluorescence spectroscopy (*λ*_ex_ = 315 nm and *λ*_em_ = 370 nm). Interestingly, we found that the homotypic condensates have the highest %EE of the 4-MUG (61.5 ± 0.8%), >2-fold higher than that of the peptide-peptide condensates (28.7 ± 2.0%) and 10-fold higher than that of the peptide-RNA condensates (5.8 ± 3.9%). EE analysis of the 4-MU product in the condensates correlates with the EE of the substrate, where the strongest partitioning is observed in the homotypic condensates (~59%), more than threefold stronger than that in the peptide-peptide condensates (~18%), and no partitioning is observed in the peptide-RNA condensates (Fig. S10).

### Spatial analysis of reaction kinetics in condensates

To spatially analyze the enzymatic reaction in the condensates, we performed confocal microscopy analysis of the reactions in the homotypic, peptide-peptide and peptide-RNA condensates. The enzyme was added to pre-formed condensates at a final concentration of 1 µg ml^−1^ and after 10 min incubation, 4-MUG was also added at a final concentration of 50 µM, and the fluorescence intensity of 4-MU was monitored over time.

**Fig. 4:**
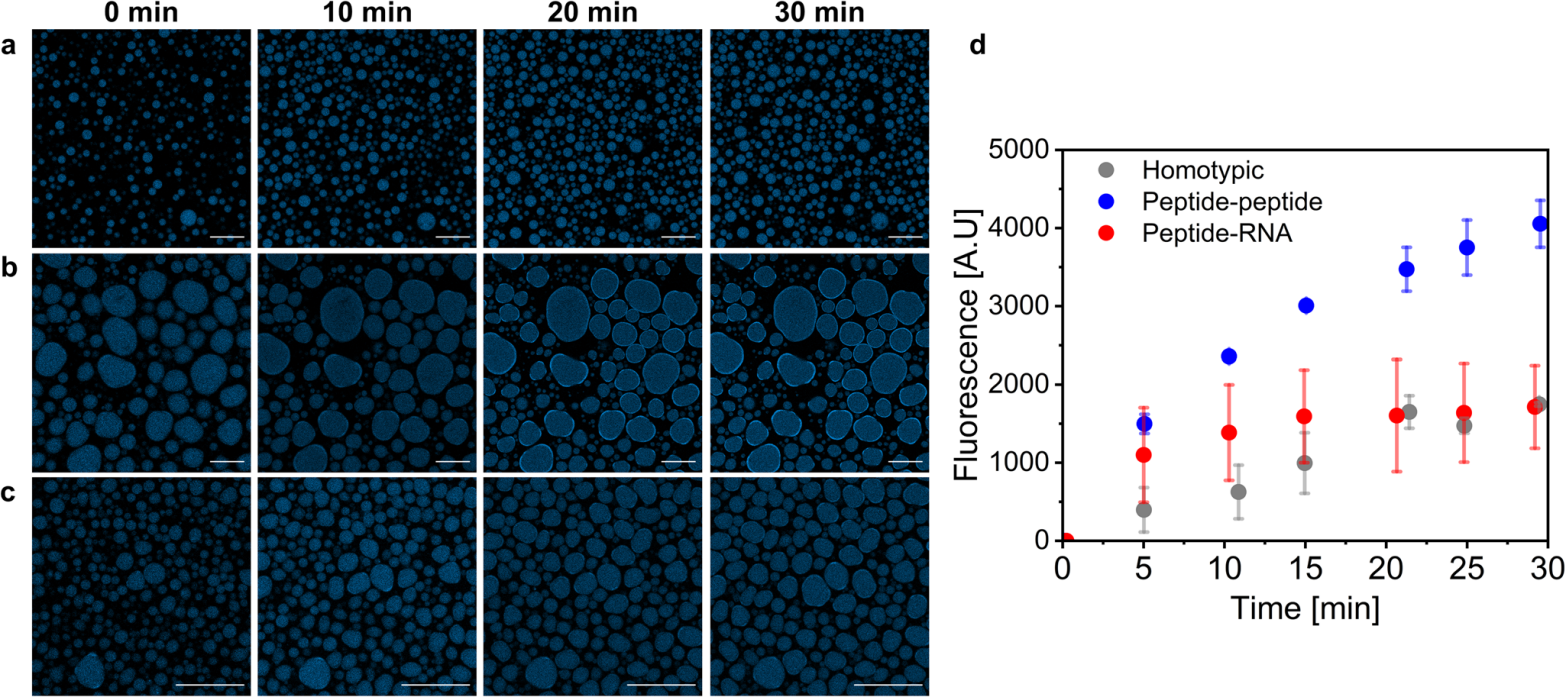
Spatial regulation of 4-MU formation in homotypic and heterotypic condensates. **a**, **b**, **c** Confocal microscopy images of 4-MU formation over time in **a** homotypic, **b** heterotypic peptide-peptide or **c** peptide-RNA condensates. Images were acquired using a *λ*_ex_ = 405 nm laser and using z-stacking analysis. All images represent the middle section of a z-stack. The transmitted light images of condensates (left) were taken at *t* = 0 min. Scale bars = 50 µm **d**. Fluorescence intensity of 4-MU in homotypic (gray), peptide-peptide (blue), or peptide-RNA (red) condensates over time, obtained by confocal microscopy analysis. Data represent the average of *N* = 30 condensates from 3 independent experiments for the heterotypic systems (10 droplets from each experiment), and *N* = 20 condensates from 2 independent experiments for the homotypic condensates. Error bars represent SD.

The highest rate of increase in 4-MU fluorescence is observed in the peptide-peptide condensates (Fig. 4b, d) and the lowest rate is observed in the homotypic condensates (Fig. 4a, d). To analyze if the reaction occurs in the dilute or condensed phase, as suggested by the confocal microscopy analysis results, we monitored the initial rates of the reaction in the dilute phase, by removing the dense phase following centrifugation and monitoring product fluorescence immediately after addition of substrate to the dilute phase. No increase in product fluorescence is observed in the dilute phase of both condensate systems (Fig. S11). Thus, this analysis suggests that the reaction does not occur in the dilute phase of either the peptide-peptide or the peptide-RNA condensates, which correlates with the high EE of the enzyme (Fig. 3a) in both systems. The results from the EE analyses, the dilute phase kinetics analysis, and the confocal microscopy analysis suggest that the restricted activity of β-gal in the homotypic condensates is not a result of limited recruitment of enzyme or substrate, but rather interference with the enzyme activity through interactions with the cationic peptide. Considering that all condensates have high enzyme EE (Fig. 3a), the findings suggest that the reaction indeed occurs in the condensed phase of all three systems, yet the differential partitioning of the product and its hydrophobicity leads to the difference observed in the confocal microscopy analysis. We presume that due to the inability of the peptide-RNA condensates to encapsulate the hydrophobic 4-MU (Fig. S10), the product is excluded from the condensed phase immediately as it forms, and therefore the fluorescence inside the peptide-RNA condensates is ~2-fold lower than that in the peptide-peptide condensates (Fig. 4, Supplementary Data 3), while the opposite trend is observed when monitoring the reaction in both phases (Fig. 2b, c). In contrast, the product is preferentially found in the condensed phase of the homotypic system, which can explain the relatively high fluorescence intensity in the homotypic condensates (Fig. 4a).

**Fig. 5:**
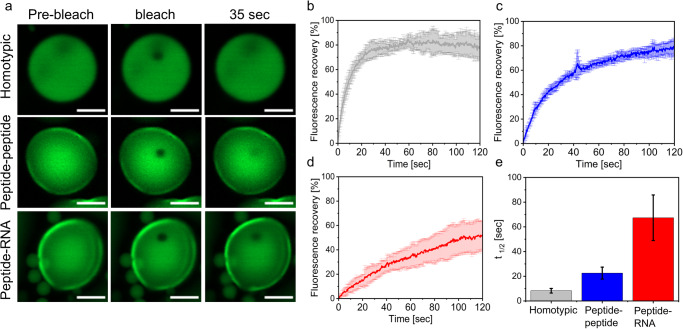
Peptide diffusivity in homotypic vs. heterotypic peptide-peptide and peptide-RNA condensates. Fluorescence recovery after photobleaching (FRAP) analysis of homotypic, heterotypic peptide-peptide and peptide-RNA condensates, performed using FITC-labeled peptides. Homotypic, peptide-peptide and peptide-RNA condensates were formed by 100 μM/20 mM, 125 μM/5 mM, and 50 μM/2 mM unlabeled/labeled peptides, respectively. **a** Confocal fluorescence images of condensates before, immediately after, and 35 sec after photobleaching. Analysis was performed using *λ*_ex_ = 488 nm laser. Scale bars = 5 µm. **b**–**d** Recovery plots from FRAP analysis of **b** homotypic, **c** heterotypic peptide-peptide and **d** peptide-RNA condensates. **e** *t*_1/2_ values calculated from recovery plots. Values of recovery plots and *t*_1/2_ represent averages of *N* = 7, 6, and 8 for homotypic, peptide-peptide, and peptide-RNA condensates, respectively. Error bars represent SD.

Next, to gain insights on the material properties of the condensates and their effect on reaction kinetics, we performed fluorescence recovery after photobleaching (FRAP) analysis of condensates using a FITC-labeled peptide. The FRAP analysis shows that the homotypic and heterotypic peptide-peptide condensates (Fig. 5a–c, Supplementary Data 4) have a similar total recovery of fluorescence (80%), while that of the peptide-RNA condensates (Fig. 5d) is significantly lower (50%). Yet, the recovery of the fluorescence represented by *t*_1/2_ in the homotypic condensates is 2.7-fold and 8-fold faster than that of the peptide-peptide and peptide-RNA condensates, respectively, with *t*_1/2_ values of 8.3 ± 1.9 s, 22.6 ± 4.8 s, and 67.3 ± 18.5 s (Fig. 5e).

### Effect of peptide hydrophobicity on reaction kinetics

**Fig. 6:**
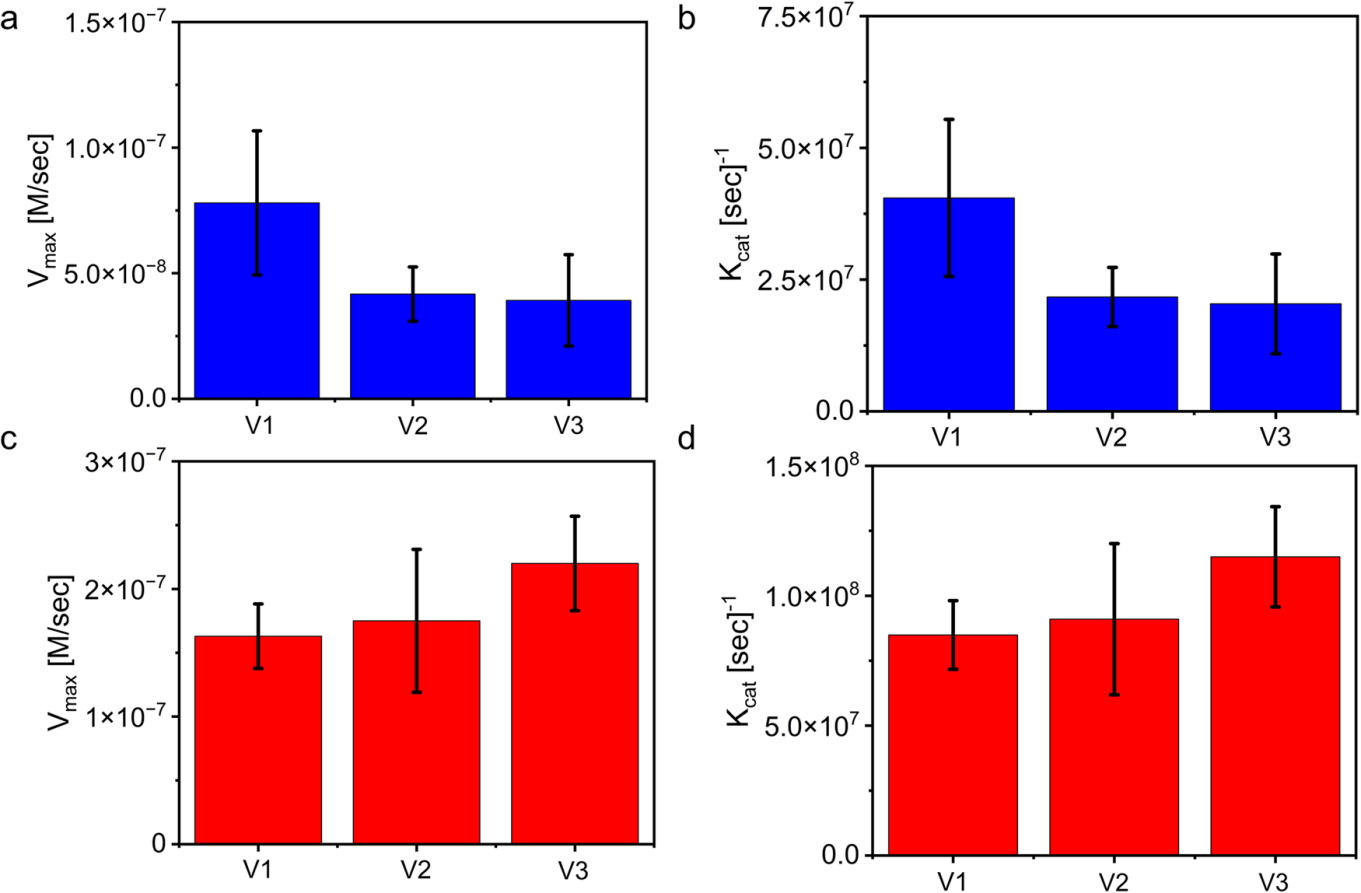
Kinetics parameters of enzymatic reaction inside condensates with varying hydrophobicity. **a**, **b** Maximum velocity (**a**) and catalytic coefficient (*K*_cat_) (**b**) of enzymatic reaction in peptide-peptide condensates formed by cationic peptides with varying hydrophobicity. According to a one-way ANOVA test, the differences between the parameters are significant. **c**, **d** Maximum velocity (**c**) and catalytic coefficient (*K*_cat_) (**d**) in peptide-RNA condensates formed by cationic peptides with varying hydrophobicity. According to a one-way ANOVA test, the differences between the parameters are not significant.

Finally, we sought to gain insights on how the chemical composition of the peptide building block, and specifically peptide hydrophobicity, affect reaction kinetics. To study peptides with varying hydrophobicity, we varied the number of valine (Val) in the peptide sequence. The primary cationic peptide (Fig. S1a) contains a single Val, and thus termed V1. We designed two additional peptides which contain two or three Val, and termed V2 and V3, respectively (peptide sequences are presented in Table S2). Heterotypic peptide-peptide and peptide-RNA condensates were formed by using either V1, V2, or V3 in complexation with the anionic peptide (Fig. S1b), or poly-U, respectively. To analyze the kinetics of β-gal activity in condensates, we monitored reactions over time by fluorescence spectroscopy at varying substrate concentrations as described above and obtained kinetic parameters for each condensate system using Michaelis–Menten and Lineweaver-Burk analysis. The kinetics analysis of the peptide-peptide condensates shows an inverse correlation between peptide hydrophobicity and *V*_max_ or *K*_cat_, where the *V*_max_ and *K*_cat_ decrease with increasing number of Val in the peptide sequence (Fig. 6a, b, Table S3, Supplementary Data 1). In contrast, increasing peptide hydrophobicity in the peptide-RNA system increases the *V*_max_ and *K*_cat_ (Fig. 6c, d, Table S3). Yet, the difference between the kinetic parameters of V1–V3 peptides are statistically significant only in the peptide-peptide system but not in the peptide-RNA condensate system, as determined by a one-way ANOVA test. These results suggest that increasing the hydrophobicity of the peptide-peptide condensates, in which the peptide concentration is higher than that of the peptide-RNA condensates, restricts the reaction, presumably due to attractive forces between the peptide(s) and the hydrophobic substrate. Similarly, hydrophobicity-dependent restriction of the enzymatic reaction is also observed in homotypic condensates, where the reaction is completely inhibited in homotypic condensates that are formed by V2 and V3 (Fig. S12).

In summary, we show that the composition of condensates affects substrate and product recruitment and the kinetics of the reaction. The homotypic system shows the highest recruitment of enzyme, substrate, and product, yet restricts the reaction. We previously showed that the interactions in such homotypic peptide condensates are mainly mediated by π-interactions between the side chains of Arg and those of aromatic amino acids^27^, and thus electrostatic interactions are not the main driving forces in this system. In contrast, the peptide-peptide and peptide-RNA systems involve complexation of cationic and anionic building blocks and are mainly driven by electrostatic interactions. Therefore, it is plausible that the protons in the homotypic condensates strongly attract the acidic side chains of the negatively charged enzyme and thus restrict its catalytic activity. The high concentration (20 mM) of the cationic peptide, which is used to form the homotypic condensates reinforces this possible explanation. Moreover, a previous study, which shows that the activity of β-gal is inhibited upon complexation with a biopolymer and regained upon complex dissociation^33^, supports this hypothesis. Our results suggest that the reaction occur in the condensed phase, yet the 4-MU product might be excluded from the peptide-RNA condensates immediately upon its formation. The limited recruitment of the hydrophobic 4-MU product in the peptide-RNA condensates is possibly a result of the polar microenvironment created by the uridylic acid functional groups. Lastly, the decrease in the kinetics in peptide-peptide condensates with increasing hydrophobicity might be a result of peptide-substrate interactions, which might, in turn, interfere with enzyme-substrate recognition and thus restrict reaction kinetics. Overall, these findings show that condensate composition and building block hydrophobicity play an important role in the regulation of enzymatic reactions and should be carefully considered in the design of phase-separated microreactors.

## Methods

### Materials

Unless otherwise specified, all reagents were of the highest available purity. Peptides were custom synthesized and purified by GenScript, Hong Kong. 4-MUG and 4-MU were purchased from Rhenium. β-galactosidase from E-coli and Atto633 protein labeling kit and poly-U were purchased from Sigma. NaCl, NaOH, and HCl were purchased from Biolab. Sodium Phosphate monobasic and Sodium Phosphate dibasic for phosphate buffer preparation were purchased from Holland Moran.

### Condensate preparation

#### Homotypic system

V1 peptide was dissolved in 36 mM phosphate buffer pH = 7.5 to a final concentration of 20 mM, pH was adjusted to 7.5. Condensates were formed following the addition of 100 mM NaCl from a 5 M stock solution.

#### Heterotypic peptide-RNA system

V1 peptide was dissolved in 36 mM phosphate buffer pH = 7.5, pH was adjusted to 7.5, and poly-U was dissolved in ddw. Condensates were formed by mixing the two stock solutions to a final concentration of 2 mM V1 peptide and 1 mg ml^−1^ poly-U.

#### Heterotypic peptide-peptide system

V1 and WGE were dissolved separately in 36 mM phosphate buffer pH = 7.5. pH was adjusted to 7.5. Condensates were formed by mixing V1 and WGE stock solutions to a final concentration of 5 mM for each peptide. To dissolve WGE, a 4 M NaOH in ultra-pure water solution was used to raise the pH of the phosphate buffer until the WGE powder was completely dissolved in the phosphate buffer, and subsequently, the pH was adjusted to 7.5 using HCl.

### Labeling of β-galactosidase enzyme

β-galactosidase was labeled using Atto633 protein labeling kit (Sigma). The labeled enzyme was purified using a gel filtration column (included in kit). The concentration of the labeled enzyme was measured by absorbance at *λ* = 630 nm and *λ* = 280 nm using an Aligent Technologies Cary 100 UV-vis spectrophotometer, and calculated to be 0.24 mg ml^−1^ using Equation (1):

1 $${C}_{{protein}}\left[{mg}{{ml}}^{-1}\right]=\frac{{A}_{280}-(0.06\times {A}_{630})}{{\varepsilon }_{{protein}}}\times {{MW}}_{{protein}}\times {Dilution\; factor}$$

Where A_280_ is the absorbance at 280 nm and A_630_ is the absorbance at 630 nm.

The labeled enzyme was freeze-dried and concentrated to a concentration of 1 mg ml^−1^.

### EE of β-galactosidase

Condensates in each system were prepared as described above. The labeled enzyme, dissolved in phosphate buffer pH = 7.5 was added to pre-formed condensates at a final concentration of 0.05 mg ml^−1^. The fluorescence signal of the enzyme was collected using a Zeiss laser scanning confocal microscope (LSM) 900 inverted confocal microscope using the *λ*_ex_ = 640 nm laser. Fluorescence intensity inside the condensates and at the dilute phase was obtained on *n* = 7 condensates and *n* = 7 dilute phase ROI from each image of the systems, experiments were performed in triplicated. The EE was calculated using Equation (2):

2 $$\% {EE}=\left(\frac{{{Intensity}}_{{droplets}}}{{{Intensity}}_{{droplets}}+{{Intensity}}_{{background}}}\right)\times 100$$

### Enzyme distribution in condensates

Condensates in each system with encapsulated β-gal enzyme were prepared as described in the previous section. The fluorescence signal of the enzyme was collected using a Zeiss LSM 900 inverted confocal microscope using the *λ*_ex_ = 640 nm laser. The fluorescent signal of a droplet from each condensates system was measured throughout 10 μm across the droplet. The condensates solutions were imaged at 1 μm Z-stacks, and the Z-stack were the droplets had the highest fluorescence was analyzed.

### EE of 4-MUG and 4-MU

A calibration curve of 4-MUG was obtained by measuring the fluorescence of the substrate at varying substrate concentrations: 5 µM, 10 µM, 25 µM, 50 µM, and 75 µM. 4-MUG dissolved in 36 mM phosphate buffer pH = 7.5. Fluorescence spectroscopy was measured using BioTek H1 synergy plate reader at *λ*_ex_ = 315 nm *λ*_em_ = 370 nm. The calibration curve was plotted as a linear fitting of the fluorescence intensity at *λ*_em_ = 370 nm as a factor of 4-MUG concentration. Then, a stock solution of 0.5 mM 4-MUG was prepared in a 36 mM phosphate buffer pH = 7.5. Condensates were formed as described above, and 4-MUG was subsequently added to a final concentration of 50 µM. In the control solution, the buffer was added to the condensates instead of 4-MUG. Following 10 min of incubation, the solutions were centrifuged for 15 min at 1000 rcf (except for the peptide-RNA condensates which were centrifuged at 20,000 rcf). After centrifugation, pellets containing the condensates and encapsulated 4-MUG were observed at the bottom of each tube, and the supernatant of the solutions was collected. The fluorescence of the supernatants was measured in a 384-well black plate with a clear bottom using a BioTek H1 synergy plate reader at *λ*_ex_ = 315 nm and *λ*_em_ = 370 nm. The concentration of 4-MUG in supernatants was calculated using the calibration curve. Each experiment was performed in triplicates.

EE was calculated using Equation (3):

3 $$\% {{{{{\rm{EE}}}}}}=\left(\frac{{C}_{i}-{C}_{\sup }}{{C}_{i}}\right)\times 100$$

When *C*_i_ is the concentration of the substance examined before centrifuge, and *C*_sup_ is the concentration of the substance in the supernatant.

EE measurements of 4-MU were similar to those of 4-MUG, except using a 50 μM 4-MU stock of the product diluting it to a final concentration of 5 μM in the condensate solution.

### Reaction kinetics analysis

For each condensate system, β-gal was added to pre-formed condensates at a final concentration of 1 µg ml^−1^ (1.92 nM). After 10 min incubation, 4-MUG was added at a final concentration of 50 µM. Product formation was monitored over time in a 384-well black plate with clear bottom by fluorescence spectroscopy using a BioTekH1synergy plate reader at ${{{{{{\rm{\lambda }}}}}}}_{{{{{{\rm{ex}}}}}}}=320\;{{{{{\rm{nm}}}}}},{{{{{{\rm{\lambda }}}}}}}_{{{{{{\rm{em}}}}}}}=450.$

### Reaction kinetics of peptide- peptide condensates with different peptide ratios

V1 and WGE were dissolved separately in 36 mM phosphate buffer pH = 7.5. pH was adjusted to 7.5. Three different condensates solutions were formed by mixing V1 and WGE stock solutions to a final concentration of 5 mM V1 and 5 mM WGE, 5 mM V1 and 2.5 mM WGE, and 2.5 mM V1 and 5 mM WGE. β-gal and 4-MUG were added to each solution as described above. Product formation was monitored over time in a 384-well black plate with clear bottom by fluorescence spectroscopy using a BioTekH1synergy plate reader at *λ*_ex_ = 320 nm, *λ*_em_ = 450.

### Reaction kinetics of dilute phase

Heterotypic peptide-peptide and peptide-RNA condensates were prepared as described above. β-gal was added to the pre-formed condensates at a final concentration of 1 µg ml^−1^ (1.92 nM). After 10 min incubation with the enzyme, the solutions were centrifuged for 2 min at 20,000 rcf. After centrifugation, the supernatant of the solutions was collected, and a 4-MUG was added to the supernatant at a final concentration of 50 μM. The reactions pre-formed at the supernatants were compared to reactions in uncentrifuged solutions. Product formation was monitored over time in a 384-well black plate with clear bottom by fluorescence spectroscopy using a BioTekH1synergy plate reader at *λ*_ex_ = 320 nm, *λ*_em_ = 450.

### Reaction kinetics below the saturation concentration of oppositely charged peptides

V1 and WGE were dissolved separately in 36 mM phosphate buffer pH = 7.5 for a concentration of 1 mM, pH was adjusted to 7.5. β-gal and 4-MUG were added to each solution as described above. Product formation was monitored over time in a 384-well black plate with clear bottom by fluorescence spectroscopy using a BioTekH1synergy plate reader at ${{{{{{\rm{\lambda }}}}}}}_{{{{{{\rm{ex}}}}}}}=320\;{{{{{\rm{nm}}}}}},{{{{{{\rm{\lambda }}}}}}}_{{{{{{\rm{em}}}}}}}=450.$

### Michaelis–Menten kinetics analysis

For a calibration curve of 4-MU, the product was dissolved at 50 µM in 36 mM phosphate buffer pH = 7.5. The fluorescence of 4-MU at varying concentration (5 µM, 10 µM, 20 µM, 30 µM, 40 µM, and 50 µM) was measured in a 384-well black plate with a clear bottom using BioTek H1 synergy plate reader at *λ*_ex_ = 320 nm, *λ*_em_ = 450 nm. The calibration curves were obtained by plotting the intensity of fluorescence at *λ*_em_ = 450 nm as a function of concentration. Due to the quenching of the product fluorescence in condensates, we obtained a separate calibration curve for each system (heterotypic peptide-peptide and peptide-RNA). Calibration curves in condensates were obtained as detailed above where the product was added to pre-formed condensates before its fluorescence was measured.

For each condensate system with each of the *V* peptides, we calculated the maximum velocity (*V*_max_), turnover number (*K*_cat_), Michaelis constant (*K*_M_), and catalytic efficiency (*K*_cat_/*K*_M_) based on Lineweaver-Burk plots. We performed a one-way ANOVA test (*p* = 0.05) using OriginLab 9.95 to analyze the significance of the differences in parameters between each system and the different peptides.

### Confocal microscopy analysis of reaction kinetics

Condensates were prepared as described above. β-gal was added at a final concentration of 1 µg ml^−1^ to solutions of pre-formed condensates. After 10 min of incubation with the enzyme, 4-MUG was added at a final concertation of 50 µM. For control solutions, condensates were not treated with enzyme. A 50 µl of the reactions were transferred to a black with a clear glass bottom 96-well-plate, glass 1.5H (produced by Hangzhou Xinyou, and purchased from Danyel Biotech), coated with 100 µl of Pluronic F-127 surfactant, dissolved in ultra-pure water at a 10 mg/ml concentration. The reaction was monitored over time for 30 min using a Zeiss LSM 900 inverted confocal microscope, using an *λ*_ex_ = 405 nm laser, and collection emission range of *λ*_em_ = 410–583 nm. Z-stacking was applied using 10 µm-width stacks. 20 condensates of each sample were analyzed using the Zen blue 3.2 software (Zeiss) to show the average fluorescence in condensates over time.

### FRAP analysis

FRAP experiments were performed using a Zeiss 900 LSM confocal microscope by tracking the fluorescent signal of FITC-labeled peptides. For the homotypic system, we used 0.1 mM FITC-labeled and 20 mM unlabeled peptides. For the peptide-peptide systems, we used 0.125 mM FITC-labeled 5 mM unlabeled peptides, and for the peptide-RNA system we used 0.05 mM FITC-labeled and 2 mM unlabeled peptide. All the solutions were transferred to a slide coated by a solution of Pluronic F-127 surfactant, dissolved in ultra-pure water at a concentration of 10 mg/ml. Photobleaching was performed using 17 iterations of *λ*_ex_ = 488 nm laser at 100% intensity, and subsequent recovery of the fluorescence at the bleached area was recorded and analyzed Zen Blue 3.2 software. Photobleaching correction and recovery time were calculated using OriginLab 9.95. The final FRAP recovery curve is the average of recovery curves collected from *N* = 6–8 separate condensates. For photobleaching correction, the emission intensity at the region of interest before photobleaching was set as the maximum (100% recovery) and the intensity immediately after photobleaching as the minimum (0% recovery).

### Reporting summary

Further information on research design is available in the Nature Portfolio Reporting Summary linked to this article.

### Supplementary information

Supplementary Information

Description of Additional Supplementary Files

Supplementary Data 1

Supplementary Data 2

Supplementary Data 3

Supplementary Data 4

Reporting Summary

## Data Availability

The authors declare that the data supporting the findings of this study are available within the paper and its Supplementary Information files, i.e., Supplementary Data 1–4. Should any raw data files be needed in another format they are available from the corresponding author upon reasonable request. Source data are provided with this paper.
