# Supplementary material for: Regulation of enzymatic reactions by chemical composition of peptide biomolecular condensates
Source: Commun Chem. 2024 Apr 20;7:90. doi: 10.1038/s42004-024-01174-7 (PMC11032315; doi:10.1038/s42004-024-01174-7)
Supplement: Supplementary file 1 — Supplementary Information [file 42004_2024_1174_MOESM1_ESM.pdf]

Supplementary Information

Regulation of enzymatic reactions by chemical composition of peptide biomolecular condensates

Rif Harris[1], Shirel Veretnik[1], Simran Dewan[1], Avigail Baruch Leshem[1] and Ayala Lampel[1-4]

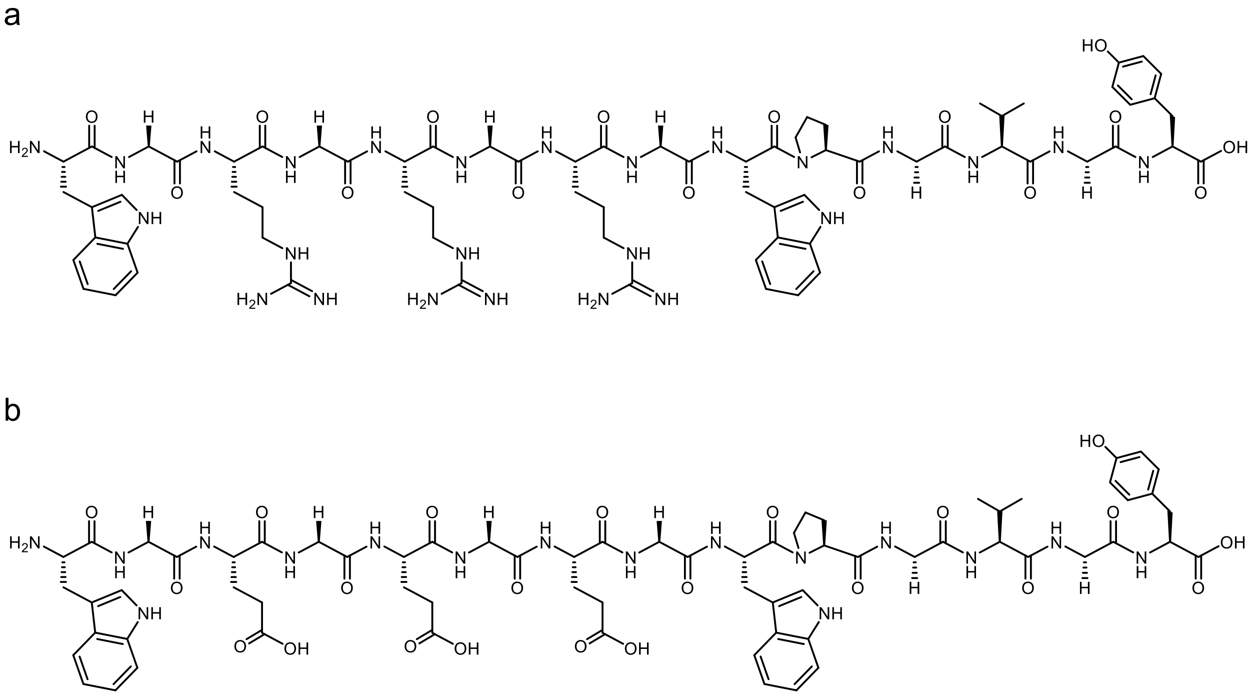

Supplementary Figure S1.**a.** Chemical structure of V1 peptide. **b.** Chemical structure of WGE peptide

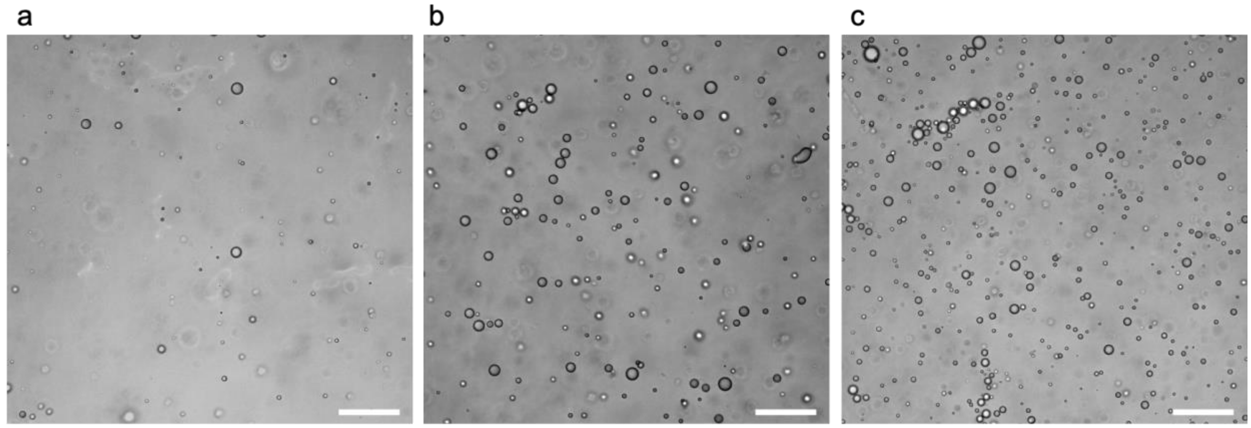

Supplementary Figure S2. Microscopy analysis of (a) homotypic, (b) heterotypic peptide-peptide and (c) heterotypic peptide-RNA condensates. Scale bars=50 μm.

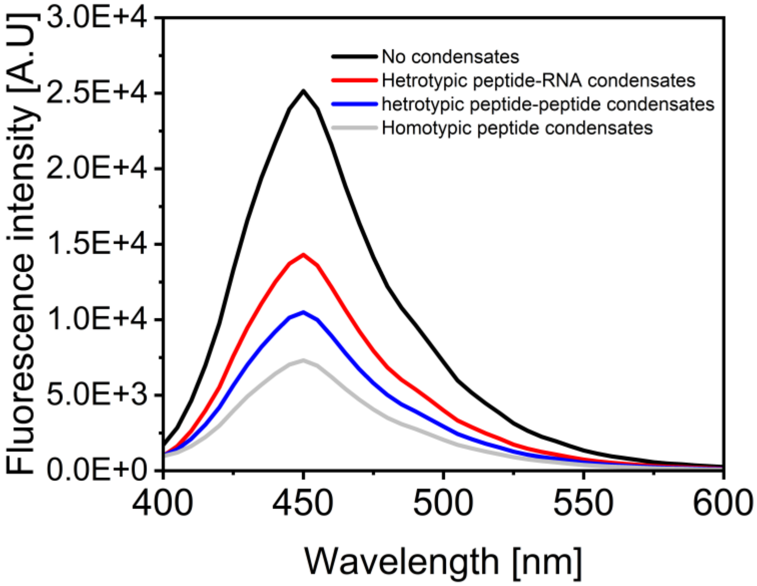

Supplementary Figure S3. Fluorescence spectra of the 4-MU product (50 μM) either in bulk (black) or in homotypic peptide (grey), heterotypic peptide-peptide (blue); or heterotypic peptide-RNA (red) condensates showing quenching of the product fluorescence in condensates.

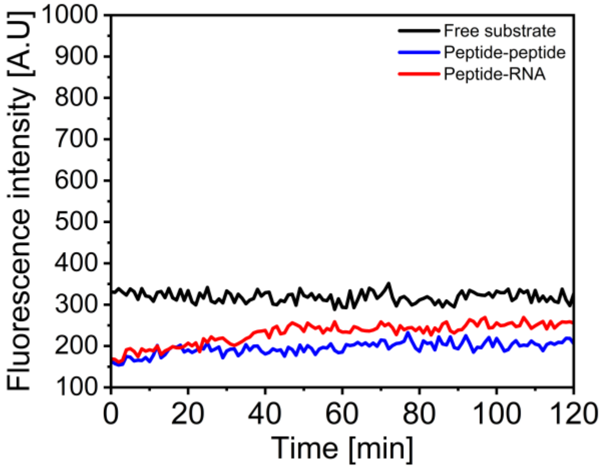

Supplementary Figure S4. Fluorescence intensity at 450 nm over time of 4-MUG in buffer (black), in peptide-peptide condensates (blue) and in peptide-RNA condensates (red).

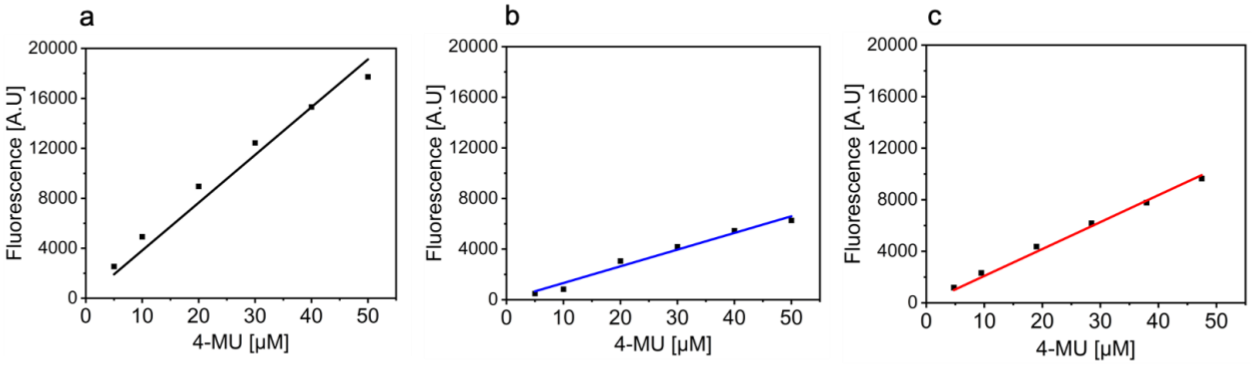

Supplementary Figure S5. Calibration curves of the 4-MU product in (**a**) free enzyme; (**b**) heterotypic peptide-peptide, and (**c**) peptide-RNA condensates.

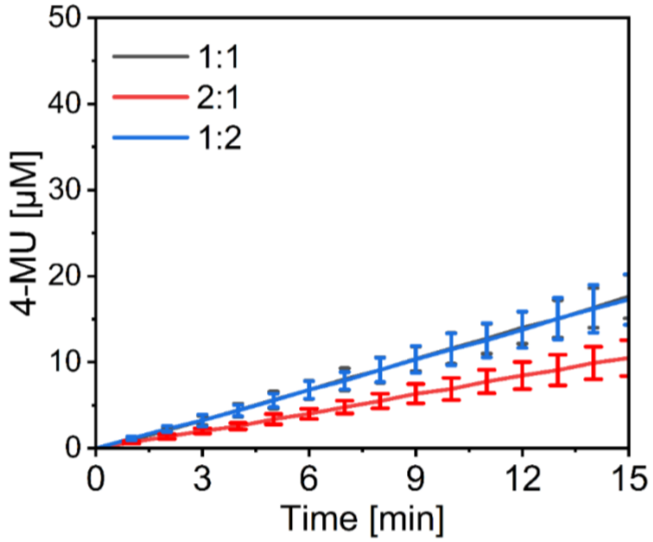

Supplementary Figure S6. Kinetics of product formation in heterotypic peptide-peptide systems at 1:1 (black), 2:1 (red) or 1:2 (blue) stoichiometry of the cationic:anionic peptides, V1:WGE. Values represent average of 3 independent experiments. Error bars represent SD.

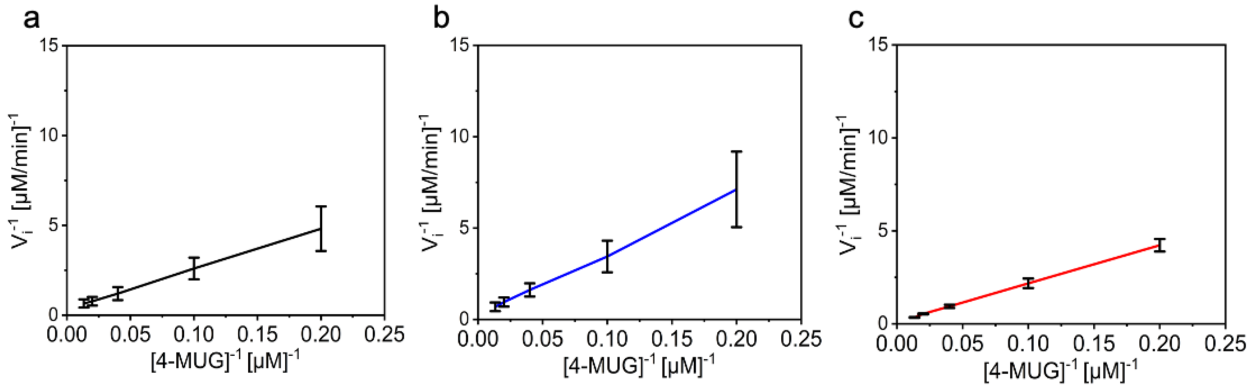

Supplementary Figure S7. Lineweaver-Burk analysis of the reaction in (**a**) buffer, (**b**) heterotypic peptide-peptide or (**c**) peptide-RNA condensates.

Table S1. Summary of kinetic parameters of free enzymatic reaction and reaction in peptide-peptide or peptide-RNA condensates

**Variable***	**Free enzyme**	**Peptide-peptide**	**Peptide-RNA**
V_{max} [M/sec]	$5.00 \pm 0.24(\times 10^{-8})$	$7.80 \pm 2.87(\times 10^{-8})$	$16.3 \pm 2.53(\times 10^{-8})$
K_{cat} $[sec]^{-1}$	$2.60 \pm 0.13(\times 10^{7})$	$4.05 \pm 1.49(\times 10^{7})$	$8.49 \pm 1.32 (\times 10^{7})$
K_m [M]	$6.73 \pm 0.33(\times 10^{-5})$	$16.0 \pm 5.89(\times 10^{-5})$	$20.2 \pm 3.14(\times 10^{-5})$
ε $[sec*M]^{-1}$	$3.86 \pm 0.27(\times 10^{11})$	$2.54 \pm 1.32(\times 10^{11})$	$4.20 \pm 0.92(\times 10^{11})$

*The kinetic parameters were obtained based on Michaelis Menten model and using fluorescence spectroscopy analysis at λ_{ex}=320 nm, λ_{em}=450 nm. The kinetic parameters of the different systems are not statistically significant based on a one-way ANOVA test.

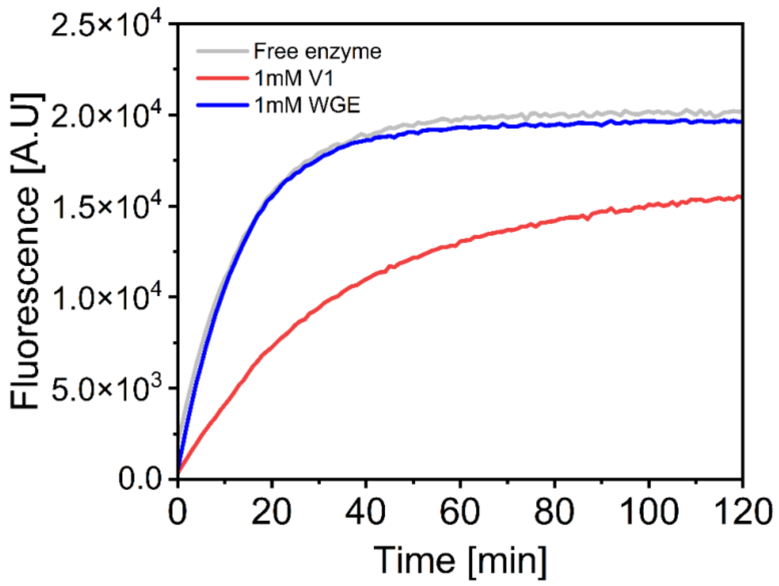

Supplementary Figure S8. Formation of 4-MU by β-gal either in the absence of condensates (grey) or in the presence of sub-saturation concentration (1 mM) of V1 (red) or WGE (blue), measured by fluorescence at 450 nm over time.

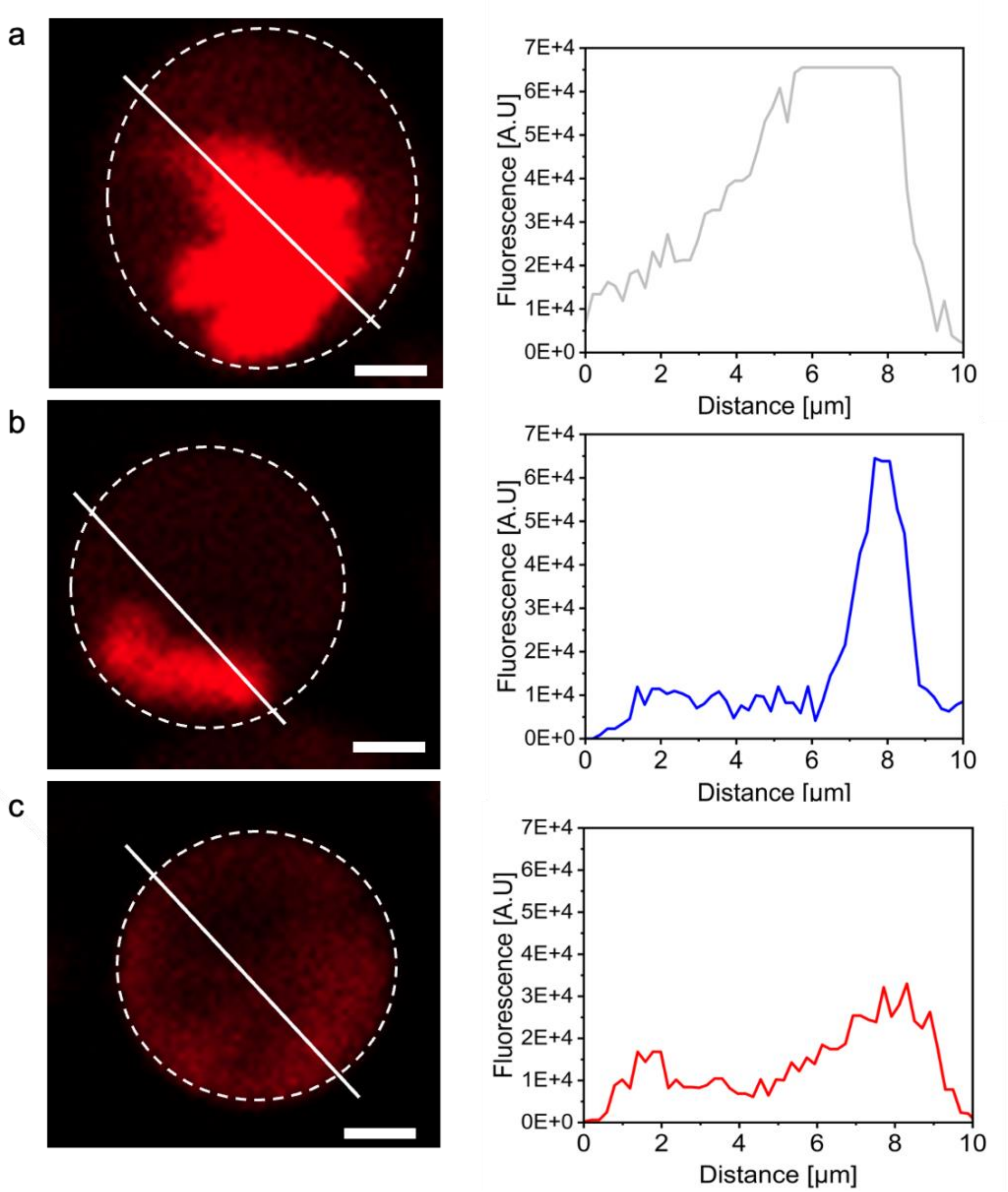

Supplementary Figure S9. **Enzyme distribution in condensates.** Confocal microscopy analysis of Atto633-labeled enzyme in **(a)** homotypic, **(b)** peptide-peptide and **(c)** peptide-RNA condensates. Images show the middle z-stack and cross-section fluorescence intensity of Atto633-β-gal. Scale bars=2 µm.

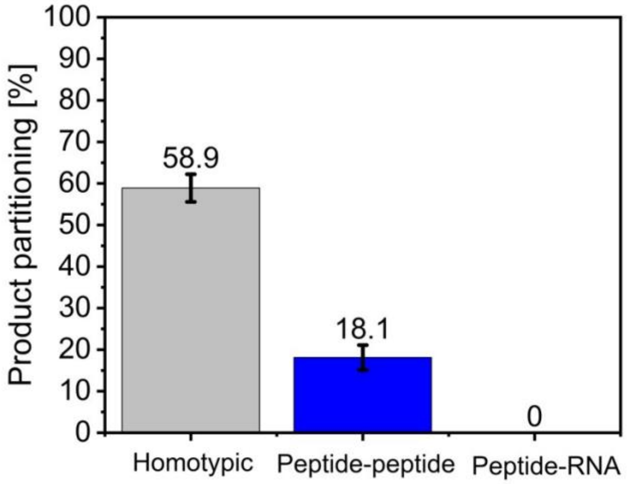

Supplementary Figure S10. Product partitioning (%) in homotypic (grey), peptide-peptide (blue) and peptide-RNA (red) condensates.

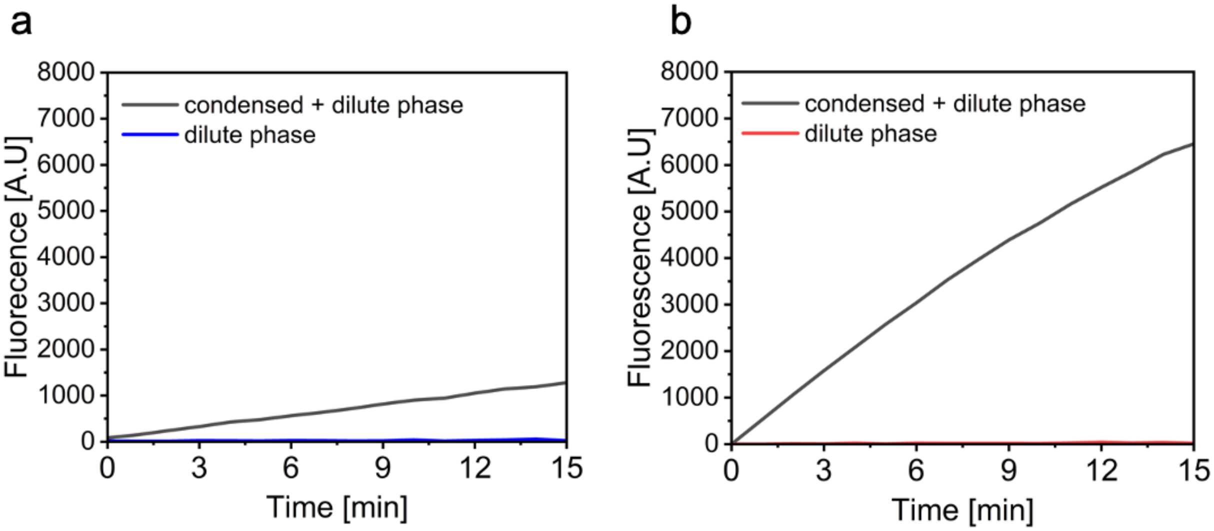

Supplementary Figure S11. Reaction in dilute phases of (**a**) peptide-RNA solution and (**b**) peptide-peptide solution.

Table S2. List of peptides which contain varying number of Val.

Peptide	Sequence
V1	WGRGRGRGWPGVGY
V2	WVRGRGRGWPGVGY
V3	WVRGRGRVWPGVGY

Table S3. Summary of kinetic parameters of different condensate systems formed by either V1, V2, or V3.

Peptide	Variable*	Peptide-peptide	Peptide-RNA
V1	V_{max} [M/sec]$_{\mathbf{ax}}$ **[M/se]**	7.80 ± 2.87(×10^{-8})	16.3 ± 2.53(×10^{-8})
	K_{cat} $[sec]^{-1}$	4.05 ± 0.67(×10^{7})	8.49 ± 1.32(×10^{7})
	K_m [M]	16.0 ± 5.89(×10^{-5})	20.2 ± 3.14(×10^{-5})
	ε $[sec*M]^{-1}$	2.54 ± 1.32(×10^{11})	4.20 ± 0.92(×10^{11})
V2	V_{max} [M/sec]	4.17 ± 1.08(×10^{-8})	17.5 ± 5.60(×10^{-8})
	K_{cat} $[sec]^{-1}$	2.17 ± 0.56(×10^{7})	9.10 ± 2.91(×10^{7})
	K_m [M]	9.04 ± 2.35(×10^{-5})	16.8 ± 5.39(×10^{-5})
	ε $[sec*M]^{-1}$	2.40 ± 0.88(×10^{11})	5.41 ± 2.45(×10^{11})
V3	V_{max} [M/sec]	2.44 ± 0.28(×10^{-8})	21.5 ± 3.06(×10^{-8})
	K_{cat} $[sec]^{-1}$	1.27 ± 0.15(×10^{7})	11.2 ± 1.59(×10^{7})
	K_m [M]	6.58 ± 0.77(×10^{-5})	19.5 ± 2.78(×10^{-5})
	ε $[sec*M]^{-1}$	1.93 ± 0.32(×10^{11})	5.74± 1.15(×10^{11})

*The kinetic parameters were obtained based on Michaelis Menten model and using fluorescence spectroscopy analysis at λ_{ex}=320 nm, λ_{em}=450 nm. The difference between the kinetics parameters of each peptide-peptide system is statistically significant; the difference between the kinetic parameters of each peptide-RNA system is not statistically significant based on a one-way ANOVA test.

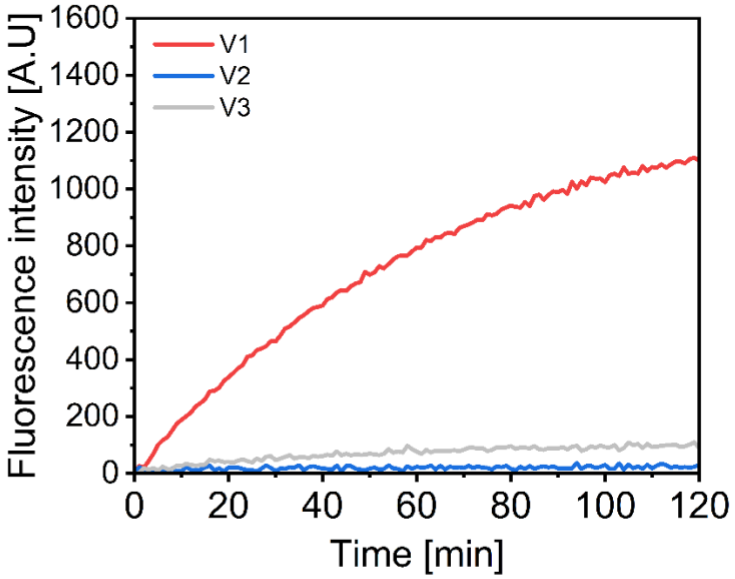

Supplementary Figure S12. Formation of 4-MU by β-gal in different homotypic condensates with varying hydrophobicity, containing either 1 (red), 2 (blue) or 3 (grey) Val, measured by fluorescence at 450 nm over time.
