# Supplementary material for: Regulation of enzymatic reactions by chemical composition of peptide biomolecular condensates
Source: Commun Chem. 2024 Apr 20;7:90. doi: 10.1038/s42004-024-01174-7 (PMC11032315; doi:10.1038/s42004-024-01174-7)
Supplement: Supplementary file 2 — Description of Additional Supplementary Files [file 42004_2024_1174_MOESM2_ESM.pdf]

Supplementary Data 1. a. Initial reaction rate and kinetic parameters of the free enzymatic reaction without condensates. **b**. Initial reaction rate and kinetic parameters of the peptide-peptide condensates formed by V1/WGE LLPS. **c**. Initial reaction rate and kinetic parameters of peptide-RNA condensates formed by V1/RNA LLPS. **d**. Initial reaction rate and kinetic parameters of the peptide-RNA condensates formed by V2/RNA LLPS. **e**. Initial reaction rate and kinetic parameters of the peptide-peptide condensates formed by V2/RNA LLPS. **f**. Initial reaction rate and kinetic parameters of the peptide-peptide condensates formed by V2/WGE LLPS. **g**. Initial reaction rate and kinetic parameters of the peptide-peptide condensates formed by V3/WGE LLPS.

Supplementary Data 2. a. Encapsulation efficiency (EE) of Atto633-labeled β-gal in homotypic condensates. **b**. EE of Atto633-β-gal in peptide-peptide condensates. **c**. EE of Atto633-β-gal in peptide-RNA condensates. **d-j**. EE of 4-MUG in the different condensates, which was analyzed by fluorescence spectroscopy at λ_{ex}=315 nm and λ_{em}=370 nm. **d**. EE of 4-MUG in homotypic condensates. **e-g**. 3 independent analyses of the EE of 4-MUG in peptide-peptide condensates. **h-j.** 3 independent analyses of the EE of 4-MUG in peptide-RNA condensates.

Supplementary Data 3. Fluorescence intensity of 4-MU in (**a**) homotypic; (**b**) peptide-peptide and (**c**) peptide-RNA condensates over time, obtained by confocal microscopy analysis.

Supplementary Data 4. Fluorescence recovery after photobleaching (FRAP) analysis of homotypic, heterotypic peptide-peptide and peptide-RNA condensates, performed using FITC-labeled peptides. Homotypic (**a**), peptide-peptide (**b**) and peptide-RNA (**c**) condensates were formed by 100 μM/20 mM, 125 μM/5 mM, and 50 μM/2 mM unlabeled/labeled peptides, respectively.
